# Saccharin Stimulates Insulin Secretion Dependent on Sweet Taste Receptor-Induced Activation of PLC Signaling Axis

**DOI:** 10.3390/biomedicines10010120

**Published:** 2022-01-06

**Authors:** Joan Serrano, Nishita N. Meshram, Mangala M. Soundarapandian, Kathleen R. Smith, Carter Mason, Ian S. Brown, Björn Tyrberg, George A. Kyriazis

**Affiliations:** 1 Department of Biological Chemistry and Pharmacology, College of Medicine, The Ohio State University, Columbus, OH 43210, USA; Joan.Serrano@osumc.edu (J.S.); meshram.8@buckeyemail.osu.edu (N.N.M.); Carter.Mason@osumc.edu (C.M.); Ian.Brown@osumc.edu (I.S.B.); 2 Sanford Burnham Prebys Medical Discovery Institute, Lake Nona, FL 32827, USA; mangalasounder@gmail.com (M.M.S.); Kathleen.smith@pfizer.com (K.R.S.); 3 Department of Physiology, Sahlgrenska Academy, University of Gothenburg, 405 30 Gothenburg, Sweden; bjorn.kiros@outlook.com

**Keywords:** saccharin, artificial sweeteners, sweet taste receptors, T1R2, T1R3, PLC, TRPM5, calcium, Munc13-1, insulin secretion, beta cells, glucose tolerance, Tdtomato, T1r2-Cre

## Abstract

Background: Saccharin is a common artificial sweetener and a bona fide ligand for sweet taste receptors (STR). STR can regulate insulin secretion in beta cells, so we investigated whether saccharin can stimulate insulin secretion dependent on STR and the activation of phospholipase C (PLC) signaling. Methods: We performed in vivo and in vitro approaches in mice and cells with loss-of-function of STR signaling and specifically assessed the involvement of a PLC signaling cascade using real-time biosensors and calcium imaging. Results: We found that the ingestion of a physiological amount of saccharin can potentiate insulin secretion dependent on STR. Similar to natural sweeteners, saccharin triggers the activation of the PLC signaling cascade, leading to calcium influx and the vesicular exocytosis of insulin. The effects of saccharin also partially require transient receptor potential cation channel M5 (TRPM5) activity. Conclusions: Saccharin ingestion may transiently potentiate insulin secretion through the activation of the canonical STR signaling pathway. These physiological effects provide a framework for understanding the potential health impact of saccharin use and the contribution of STR in peripheral tissues.

## 1. Introduction

Artificial sweeteners (AS) are food additives that enhance sweet flavor without an extra energy burden. Consequently, their use has increased considerably over the past decade [1] by those intending to limit calories or control blood glucose. However, some AS may not be physiologically inert as originally thought. For instance, the consumption of saccharin in mice and healthy humans was shown to induce glucose intolerance through changes in gut microbiota [2], but these findings could not be subsequently replicated [3]. Moreover, saccharin supplementation did not induce glucose intolerance in healthy overweight or obese individuals [4], nor did it change fasting glucose and insulin in patients with type 2 diabetes [5]. These findings do not preclude possible effects of saccharin use, but understanding whether and how acute saccharin ingestion triggers the physiological process may be imperative for shedding light on its chronic effects.

For instance, saccharin induces significant amplifying effects on insulin secretion in mouse and human islets [6,7,8,9], suggesting potential acute physiological effects in the regulation of glucose homeostasis. Although the mechanism is still unknown, it has been assumed that STR are the de facto mediators because these G-protein-coupled receptors (GPCRs) are expressed in pancreatic beta-cells and saccharin is a bona fide ligand. Using genetic and pharmacological approaches, we previously showed that fructose potentiates insulin secretion dependent on STR and the activation of a PLC signaling cascade [9]. However, similar to other GPCRs, there is evidence suggesting that STR may have diverse signaling partners depending on the ligand and cell context [10]. Therefore, we set to investigate whether saccharin-induced insulin secretion is dependent on STR and elucidate the signaling mechanism.

## 2. Materials and Methods

### 2.1. Reagents and Animals

All chemicals and cell culture reagents were purchased from Sigma Aldrich (St Louis, MO, USA) or Invitrogen (Carlsbad, CA, USA), unless otherwise specified. Mice with the homozygous deletion for the T1r2 [11] or Trpm5 [12] gene (kindly provided by Dr Zuker, Columbia University) were back crossed on the C57Bl\6J strain for at least 10 generations, bred, and genotyped in-house. Age-matched (10–14 weeks) male mice with littermate controls were cohoused and randomly assigned to experimental groups. Mice had similar body weights (23–25 g) and were maintained on the same chow diet. All animals were maintained in accordance with the National Institutes of Health Guidelines for the Care and Use of Laboratory Animals, and all experiments were performed with approved protocols from the Institutional Animal Care and Use Committee of the Ohio State University (2018A00000039-R1; Approval 20 April 2021).

### 2.2. T1r2-Cre Knock-in (KI) Mouse

The Tas1r2-Cre KI mouse strain was created by the Ingenious Targeting Laboratory, (Ronkonkoma, NY, USA). A 7.9 kb region used to construct the targeting vector was first sub cloned from a positively identified C57BL/6 fosmid clone (WI1-55G11) using a homologous recombination-based technique. The region is designed such that the 5′ homology arm extends 5.8 kb upstream of the Cre-pA KI cassette, and the 3′ homology arm extends 2.0 kb downstream of the Neo cassette. The Cre-pA KI cassette is inserted immediately downstream of the ATG start site of the mouse Tas1r2 gene. The Neo selection cassette flanked by FRT sites is followed immediately downstream of the Cre-pA KI cassette. The targeting vector was confirmed by restriction analysis after each modification step. The boundaries of both homology arms were confirmed by sequencing with P6 and T73 primers that read through both sides of the backbone vector into the genomic sequence. The junctions of the Neo cassette and the entire Cre-pA KI cassette were confirmed by sequencing. The targeting vector was electroporated into ES cells, and was subsequently screened ES cell clones by PCR and Southern blotting. The targeted clones were microinjected into Balb/c blastocysts and the resulting chimeras with a high percentage of black coat color were mated to C57BL/6J WT mice to generate germline Neo deleted mice. Resulting pups were genotyped and sequenced for Cre KI and a representative mouse (clone 474) was used as founder. T1r2-Cre KI mice were backcrossed into C57BL/6J strain for 10 generations.

### 2.3. T1R2 Reporter Mice

T1r2-Cre KI mice were crossed with floxed TdTomato mice (Jax# 007914, The Jackson Laboratory, Bar Harbor, ME, USA) to generate T1R2-Cre:TdTomato-fl reporter mice. T1r2 reporter mice were subsequently crossed with glucagon GFP KI mice (RBRC# 02831, Riken, Japan) to generate T1r2 and glucagon double-reporter mice. All mice were genotyped for Cre presence (Fw, AGATGCCAGGACATCAGGAACCTG; Rv, ATCAGCCACACCAGACACAGAGATC), Cre KI integration in the T1r2 allele (Fw, TCCTGCTCCAGTCTCTGTTCCTTG; T1r2-Cre Rv, CCCAGCATCCACATTCTCCTTT; T1r2-WT Rv CACGTTGGCATGGAGGGTAAA), glucagon-GFP KI (Fw, TGAGCTCATTTGGACTGCCTGC; Gcg-GFP Rv, ATGGTGCGCTCCTGGACGTAG; Gcg-WT Rv, GGGATATCAATGTAATAACCACAAACGGTA), and TdTomato (TdTom-WT Fw, AAGGGAGCTGCAGTGGAGTA; TdTom-WT Rv, CCGAAAATCTGTGGGAAGTC; TdTom-KI Fw, CTGTTCCTGTACGGCATGG; TdTom-KI Rv, GGCATTAAAGCAGCGTATCC).

### 2.4. Cell Culture

MIN6 β-cells (kind gift of Dr. Miyazaki) were cultured in DMEM containing 25 mM glucose and L-glutamine, supplemented with 15% fetal bovine serum, 100 units/mL penicillin and 100 μg/mL streptomycin, 0.25 µg/mL of amphotericin-B (Antibiotic-Antimycotic), and 5 μL/l β-mercaptoethanol under 10% CO_2_. Cells between passages 5–20 (post-arrival to the lab) were used for all experiments.

### 2.5. In Vivo Experiments

All in vivo experiments were performed with 8–10 week old male mice on regular chow (Envigo, Indianapolis, IN, USA). For the an intragastric glucose tolerance test (IGGTT), mice were fasted overnight and received a solution (~0.18 mL) of glucose (694 mM; 1 g/kg) with or without saccharin (0.1%) using a 22-gauge feeding needle (Kent Scientific, Torrington, CT, USA). Then, blood glucose or insulin was measured over time. For IP potentiation of saccharin, a bolus of saccharin (0.1%) or saline was administered in 5 h fasted mice, and blood levels of insulin were measured. For intravenous (IV) potentiation of saccharin, a bolus of saccharin (25 mg) or saline was administered exactly as described [9]. Blood glucose was sampled from the tail and analyzed with an AlphaTRAK blood-glucose-monitoring meter (North Chicago, IL, USA). Glucose tolerance curves over time are shown in absolute values. Area under curve (AUC) was calculated using the trapezoid method adjusted for fasted baselines. Insulin was analyzed with an insulin mouse ultra-sensitive ELISA kit (Crystal Chem, Grove Village, IL, USA). Rate of insulin secretion was calculated as the slope of 0–10 min and 0–15 min during the IP injection and IGGTT, respectively.

### 2.6. Static Insulin Secretion

Mouse islets were isolated and cultured exactly as described [9]. Isolated mouse islets were equilibrated in Krebs–Ringer HEPES buffer (KRH: 119 mM NaCl, 4.74 mM KCl, 2.5 mM CaCl_2_, 1.2 mM MgCl_2_, 1.2 mM KH_2_PO_4_, 10 mM HEPES, 0.5% insulin-free BSA, pH 7.4) with glucose (mouse 8.3 mM, unless otherwise stated). Islets were hand-picked and transferred to custom-made wells with glucose only for 30 min (i.e., baseline). Islets were then transferred to new wells and challenged with glucose, with or without saccharin, for additional 30 min. The supernatant from both incubations was collected and static insulin secretion was measured using ELISA (Mercodia, Uppsala, Sweden). Islet insulin secretion was expressed as absolute values (μg/L) or as fold-change of stimulated/baseline insulin secretion using paired experiments. For each experimental condition, we independently measured insulin from 4 separate wells (10 islets each), which were then averaged to represent insulin values for n = 1. All insulin secretion data are averages of n = 6–8 independent mouse islet isolations as shown. If necessary, solutions were osmotically balanced using appropriate concentrations of mannitol that have negligible affinity for STR. All islet incubations were at 37 °C without CO_2_ in 100 μL of KRH buffer.

### 2.7. Calcium Imaging

MIN6 beta-cells were plated at a density of 1 × 10^6^ cells/35mm glass bottom MatTek dish (Ashland, MA, USA) the day before the experiment. For T1r3 silencing, MIN6 beta-cells were transfected with siRNA targeting t1r3 (Invitrogen, Carlsbad, CA, USA) or scrabbled control using Lipofectamine 2000 (Thermo Fisher Scientific, Columbus, OH, USA) reagent for 48 h. The knock-down efficiency was about 50% (control 285 ± 30 vs. siT1r3 157 ± 9; n = 3 two-tailed *T*-test, *p* = 0.015). Islets were trypsinized and then dispersed in MatTek dishes pre-coated with poly-L-lysine. Dispersed islets were transfected a day prior with a construct expressing the red fluorescent protein, Katushka, under the rat insulin promoter II (RIP) to exclusively identify and image β-cells. Cells were loaded with 3 μM Fura-2 acetoxymethyl ester in KRH buffer containing 8.3 mM glucose for 20 min and then washed twice with KRH and incubated for additional 30 min at 37 °C without CO_2_. Dishes were placed into a heated chamber mounted on the stage of an inverted fluorescence microscope (Nikon Eclipse TiE with perfect focus and DG-5 Xenon excitation) and perifused with KRH pus 8.3 mM glucose (unless otherwise stated) at a rate of 1.5 mL/minute. Baseline was established for at least 6 min before stimulation, as shown in the figures. Fura-2 dual excitation images were captured through a Nikon S Fluor 20X objective (NA 0.75) with a Photometrics QuantEM 16bit EMCCD camera using 340 nm and 380 nm excitation filters and a 470–550 nm emission filter. Data were acquired and analyzed using Nikon Elements software (Nikon Instruments Inc., Melville, NY, USA). The fluorescence intensities of several single primary β-cells or 20–40 MIN6 cells per dish/condition were background subtracted and expressed as ratio of excitation 340/380 nm.

### 2.8. Total Internal Reflection (TIRF) Microscopy

MIN6 beta-cells were transfected 24 h before the experiment with a PH_PLCδ1_-GFP construct in which the PIP_2_-binding pleckstrin homology domain of PLC-δ was fused to GFP (kindly provided by Dr. Tengholm, Uppsala University, Sweden) or with a Munc13-1 fused to GFP (Addgene, Watertown, MA, USA) using Lipofectamine 2000 reagent. Growth medium was removed and replaced with KRH buffer containing 8.3 mM glucose. Similar procedures to “Calcium Imaging” section followed on the same microscope platform. Cell-surface-associated PH_PLCδ1_-GFP or Munc13-1-GFP was excited with an evanescent wave created by a 488 nm argon laser line, and GFP emission was visualized using a 500–550 nm band-pass emission filter. Images were captured using a Nikon Apo TIRF 60X oil immersion objective (NA 1.49) using a Photometrics Coolsnap HQ2 14bit CCD camera. Data were acquired and analyzed using Nikon Elements software (Nikon Instruments Inc., Melville, NY, USA). The fluorescence intensities (F) of 4–6 cells were background (F_0_) subtracted and normalized to average base-line values (ΔF − F_0_).

### 2.9. Immunofluorescence Microscopy

Pancreata were harvested from GLUC-eGFP:T1R2-Cre:Tdtomato-fl mice and fixed in 4% paraformaldehyde for 2 h and then transferred to 30% sucrose overnight at 4 °C. Pancreata embedded in OCT were cut into 8 µm thick cryostat sections (Leica, Allendale, NJ, USA) and collected onto Superfrost Plus glass slides (Thermo Fisher Scientific, Columbus, OH, USA). Heat-induced antigen retrieval was performed by microwaving in 10 mM sodium citrate buffer pH 6.0. Slides were then washed with de-ionized water for 5 min followed by wash with 1X PBS. Tissue sections were marked with PAP pen to create hydrophobic barrier (EMS, Cat. No. 71312). Sections were blocked in 5% Donkey Serum (Sigma, Ronkonkoma, NY, USA; Cat. No. D9663), 1% BSA, and triton X 100 in 1X PBST in a humid chamber at room temperature for an hour. Sections were then incubated overnight with Recombinant Anti-Insulin antibody [EPR17359] (Abcam, Waltham, MA, USA; Cat. No. ab181547) and Anti-RFP (GOAT) antibody (Rockland, Pottstown, PA, USA; Cat. No. 200-101-379) either separately or together. On the following day, sections were washed 3 times for 5 min in 1X PBS followed by incubation in anti-rabbit and anti-goat conjugated to Alexa (Jackson ImmunoResearch, West Grove, PA, USA) secondary antibodies at room temperature in the dark humid chamber. Finally, for nuclear staining, sections were counterstained in DAPI (Thermo Fisher Scientific, Columbus, OH, USA; Cat. No. EN62248) for 10 min in the dark humid chamber, briefly washed with 1X PBS for 5 min, and mounted using ProLong™ Gold Antifade Mountant (Thermo Fisher Scientific, Columbus, OH, USA; Cat. No. P36930). For controls, no primary antibody was used. Images were captured using Zeiss LSM 900 Confocal microscope and analyzed by Zen 3.3 Blue edition (Carl Zeiss Vision Inc, Whiate Plains, NY, USA). All images were captured using 20X objective and processed under identical conditions.

### 2.10. Statistical Analysis

All results are presented as mean +/− standard error of the mean (SEM). The level of significance was set at *p* < 0.05. Statistical significance was calculated as shown in figure legends.

## 3. Results

### 3.1. The T1R2 Sweet Taste Receptor Is Expressed in Beta-Cells of Mouse Islets

**Figure 1:**
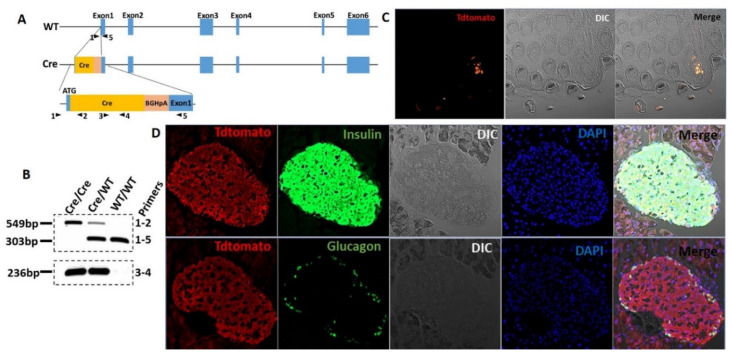
The T1R2 sweet taste receptor is expressed in beta-cells of mouse islets. (**A**) Construct development and generation of T1r2-Cre mice. (**B**) Genotyping of T1r2-Cre mice using two separate PCR reactions. (**C**) Tdtomato expression in taste buds of the tongue in T1r2-Cre:Tdtomato-fl/fl mice using fluorescent microscopy of endogenous Tdtomato. Differential interference contrast (DIC). (**D**) Co-expression of Tdtomato with glucagon or insulin in islets from T1r2-Cre:Tdtomato-fl/fl mice using immuno-fluorescent microscopy. Scale bar: 20× magnification.

Evidence for the expression of STR in isolated islets or beta-cell lines is limited to RT-qPCR [8,9], since commercially available antibodies are not reliable. To circumvent these limitations, we developed a T1r2-Cre mouse (Figure 1A,B) and subsequently crossed it with Tdtomato-fl/fl reporter mice to assess T1r2-expressing cells in mouse islets. Tdtomato was specifically expressed in select taste buds of the tongue, confirming the validity of the mouse model (Figure 1C). Tdtomato was broadly expressed in pancreatic islets, co-localizing with insulin-, but not with glucagon-positive cells (Figure 1D).

### 3.2. Saccharin Potentiates Insulin Secretion In Vivo Dependent on STR

**Figure 2:**
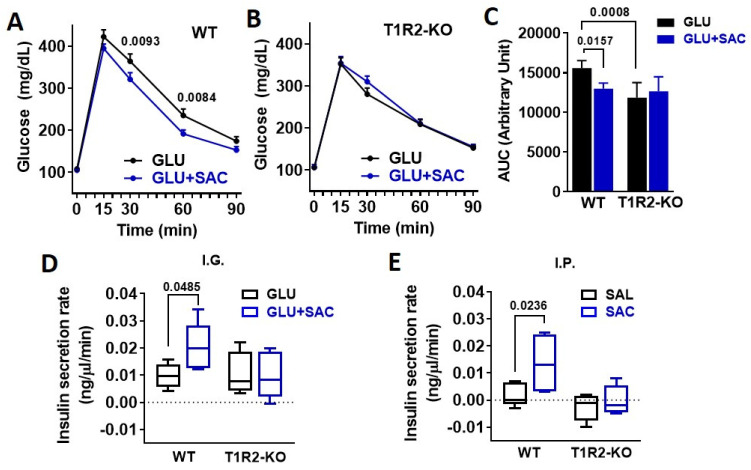
Saccharin potentiates insulin secretion in vivo dependent on STR. (**A**,**B**) Glucose excursions in WT and T1R2-KO mice in response to an IGGTT (GLU, 694 mM; 1 g/kg) with or without saccharin (SAC, 0.1%) (n = 10 mice/group). Two-way ANOVA Sidak post hoc. (**C**) Area under curve (AUC) of glucose during the IGGTT was calculated as response X time. ANOVA Fisher’s LSD post hoc. (**D**) Insulin responses in WT and T1R2-KO mice in response to an IGGTT (GLU, 694 mM; 1 g/kg) with or without saccharin (SAC, 0.1%) (n = 5 mice/group). Two-way ANOVA Fisher’s LSD post hoc. (**E**) Insulin responses in WT and T1R2-KO mice during a single IP bolus of saline (SAL) or saccharin (SAC, 0.1%) (n = 5 mice/group). Two-way ANOVA Fisher’s LSD post hoc.

To address the physiological relevance of saccharin ingestion, we performed IGGTT with or without saccharin. The addition of saccharin reduced plasma glucose excursions in WT mice (Figure 2A,C). Compared to WT, T1R2-knockout (KO) mice had reduced glucose responses during the glucose gavage (Figure 2C) as previously described [13]. However, the co-ingestion of saccharin had no further effects on glucose excursions (Figure 2B,C). Saccharin caused a moderate increase in plasma insulin in WT, but not in KO mice (Figure 2D), suggesting that the reduced IGGTT response in WT mice is likely linked to a potentiation of insulin secretion by saccharin. Due to the fact that several mechanisms can affect glucose and insulin responses to an IGGTT, we directly administered saccharin through an IP injection and assessed acute insulin responses compared to a saline control. Saccharin induced a substantial increase in plasma insulin in WT mice, but not in KO mice (Figure 2E).

### 3.3. Saccharin-Induced Signaling Is Dependent on STR and Calcium Flux

**Figure 3:**
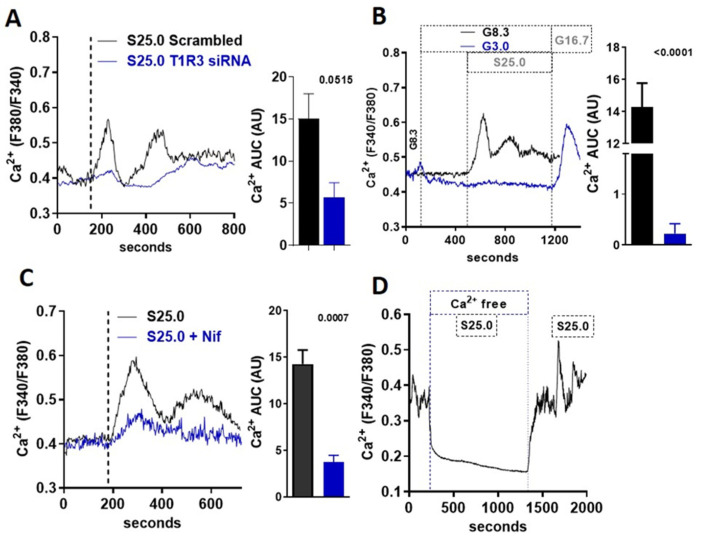
Saccharin-induced signaling in beta-cells is mediated by calcium influx. (**A**) Representative trace of intracellular calcium response in MIN6 beta-cells treated (vertical line) with saccharin (S; 25.0 mM + 8.3 mM glucose) with or without T1R3 knock-down. Inset: area under curve (AUC) calculated as response X time (n = 6). (**B**) Representative traces of intracellular calcium response in MIN6 beta-cells treated (vertical line) with saccharin (S; 25.0 mM) in the presence of 3.0 or 8.3 mM glucose (G). Area under curve (AUC) calculated as response X time (n = 4). (**C**) Representative traces of intracellular calcium response in MIN6 beta-cells treated (vertical line) with saccharin (S; 25 mM) and nifedipine (Nif; 1.0 mM) as shown. Inset shows area under curve (AUC) calculated as response X time. (**D**) Representative trace of intracellular calcium response in MIN6 beta-cells treated with saccharin (S; 25.0 mM) with or without extracellular calcium (Ca-free). All experiments show average traces of 20–40 cells (n = 4–6 per group). All panels show *p* value using two-tailed unpaired *T*-test.

An increase in the intracellular calcium concentration ([Ca^2+^]_i_) is required for the stimulation of insulin release in beta-cells. Using Fura-2-based cell imaging in MIN6 beta-cells, we tested whether saccharin could stimulate a [Ca^2+^]_i_ response at a physiological glucose concentration (8.3 mM). Saccharin induced an increase in [Ca^2+^]_i_ characterized by an initial rapid peak, followed by a second sustained response (Figure 3A). The effects of saccharin on calcium flux were dependent on STR signaling, since the moderate knock-down of the T1R3 receptor partially ablated saccharin responses (Figure 3A). These data suggest that saccharin activates STR on beta-cells to mediate [Ca^2+^]_i_ responses. STR signaling in beta-cells depends on physiological glucose concentrations [9]. Similarly, calcium responses elicited by saccharin were also dependent on glucose, since the rise in [Ca^2+^]_i_ vanished at sub-stimulatory glucose levels (3.0 mM) (Figure 3B). Due to the fact that a voltage-dependent calcium channel (VDCC) activation and calcium influx are required for glucose-stimulated insulin secretion (GSIS), we used nifedipine, a known inhibitor of VDCCs, to test whether saccharin-induced signaling depends on the activation of the extracellular calcium entry. Pretreatment with nifedipine (Figure 3C) or the removal of extracellular calcium (Ca^2+^-free) (Figure 3D) drastically reduced the second-phase calcium responses.

### 3.4. Saccharin Stimulates Insulin Secretion Dependent on PLC Activation

**Figure 4:**
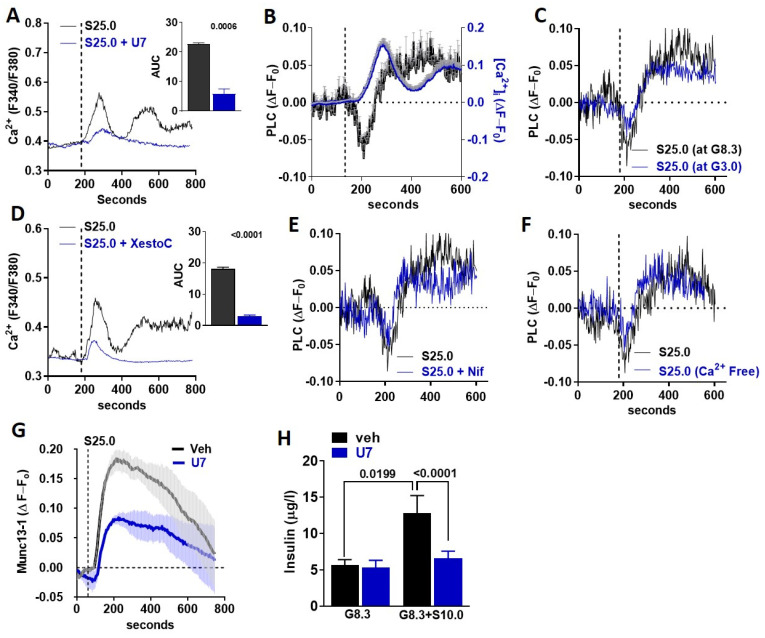
PLC activation is essential for saccharin-induced STR signaling (**A**,**D**) Representative traces of intracellular calcium response in MIN6 beta-cells treated (vertical line) with saccharin (S; 25.0 mM) with or without (**A**) U73122 (U7; 5.0 mM) and (**D**) xestospongin C (XestoC; 1.0 mM). Insets show area under curve (AUC) calculated as response X time. All experiments shown are average traces of 20–40 cells (n = 4–6 per group) and were performed in the presence of 8.3 mM glucose unless otherwise stated. Two-tailed unpaired *T*-test. (**B**) Average of traces (4–6 cells/trace total n = 6 independent experiments) showing relative change in PLC activity (left axis) or intracellular calcium (right axis) from baseline (set at 0) in MIN6 beta-cells treated (vertical line) with saccharin (25 mM) in the presence of glucose (8.3 mM). (**C**,**E**,**F**) Representative traces showing relative change in PLC activity from baseline set at 0 in MIN6 beta-cells treated (vertical line) with saccharin (S; 25 mM) in the presence of (**C**) glucose (G; 3.0 mM or 8.3 mM), (**E**) nifedipine (Nif; 1.0 mM), or (**F**) no extracellular calcium (Ca-free). Experiments were performed in the presence of 8.3 mM glucose, unless otherwise stated, and represent average of 4–6 cells. (**G**) Average of traces (4–6 cells/trace total n = 6 independent experiments) showing relative change in Munc13-1 activity from baseline (set at 0) in MIN6 beta-cells treated (vertical line) with saccharin (25 mM) in the presence of glucose (8.3 mM) with or without U73122 (U7; 5 mM). (**H**) Static insulin release in response to glucose (G; 8.3 mM) and saccharin (S; 10 mM) with or without U73122 (U7; 5.0 mM) in WT islets (n = 4–6 mice per group). ANOVA Fisher’s LSD post hoc.

STR can activate PLCβ2 [14], which is expressed in MIN6 beta-cells and mouse islets [9]. Therefore, we tested whether the canonical STR pathway is activated in beta-cells in response to saccharin. The inhibition of PLC activity with U73122 (U7) abolished saccharin-induced [Ca^2+^]_i_ responses in MIN6 beta-cells (Figure 4A). Next, we used TIRF microscopy [15] to assay the time-course of PH-PLCδ-GFP biosensor translocation [16] between the plasma membrane and the cytoplasm (i.e., PLC activity) in beta-cells, as previously described [9]. PLC translocation rapidly increased (a rapid drop in fluorescence intensity) upon saccharin stimulation (Figure 4B left axis). The activation of PLC immediately preceded the onset of the [Ca^2+^]_i_ response (Figure 4B right axis), suggesting that, similarly to fructose [9], STR activation by saccharin directly triggers PLC in beta-cells. Given that [Ca^2+^]_i_ flux was dependent on glucose availability (Figure 3B), we tested whether similar glucose requirements were necessary for PLC activation. Saccharin-induced PLC activation was similar at sub-stimulatory (3.0 mM) or normal glucose (8.3 mM) levels (Figure 4C), suggesting that PLC activation precedes all signaling requirements associated with beta-cell depolarization. PLC activation induces calcium release from the ER [14]. To test the dependency of saccharin signaling on ER calcium release, we used xestospongin C, a non-specific inhibitor of IP_3_ receptors [17]. The inhibition of ER calcium release reduced the first calcium peak and completely abolished the second prolonged phase, suggesting that ER calcium mobilization is required for initiating [Ca^2+^]_i_ responses in beta-cells (Figure 4D). Next, we tested whether PLC activation precedes the activation of VDCCs and [Ca^2+^]_i_ influx. Neither the inhibition of VDCCs with nifedipine (Figure 4E) nor the absence of extracellular calcium (Figure 4F) affected the activation of PLC in response to saccharin, further confirming that PLC activation is upstream of [Ca^2+^]_i_ influx. Munc13-1 is necessary for insulin granule fusion and insulin secretion [18,19,20,21,22,23]. We used a Munc13-1-EGFP biosensor [24] in combination with TIRF microscopy to track its appearance in the plasma membrane (i.e., indicative of granule translocation and fusion). We found that, in MIN6 beta-cells, the activation of STR by the saccharin-induced rapid translocation of Munc13-1 to the plasma membrane was dependent on PLC activity (Figure 4G). Finally, PLC inhibition diminished insulin release in isolated islets in response to saccharin, but did not alter steady-state insulin release at 8.3 mM glucose (Figure 4H). Collectively, these data suggest that saccharin induces the direct activation of the PLC-IP_3_ pathway for the regulation of insulin secretion.

### 3.5. TRPM5 Mediates the Effects of Saccharin-Induced Activation of STR Signaling in Beta-Cells

**Figure 5:**
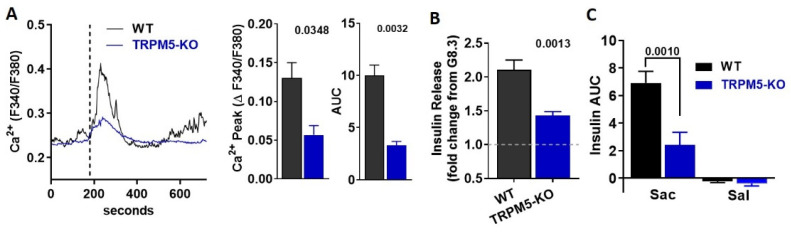
Ablation of TRPM5 abolishes the effects of saccharin in mouse islets and in vivo. (**A**) Representative traces of intracellular calcium response in WT and TRPM5-KO primary beta-cells treated (vertical line) with saccharin (25.0 mM) in the presence of 8.3 mM glucose (n = 3 mice per group; average of 5–10 cells). Insets: calcium response shown as change between baseline and peak calcium response. Area under curve (AUC) calculated as response X time. Two-tailed unpaired *T*-test. (**B**) Static insulin release in response to saccharin (20 mM) in the presence of 8.3 mM glucose in WT and TRPM5-KO islets (n = 6 mice per group). Data are expressed as fold change from 8.3 mM glucose (set at value 1) using paired experiments. Two-tailed unpaired T-test. (**C**) Plasma insulin in response to a bolus of saccharin (sac; 1.0 g/kg) or saline (sal) injected in WT and TRPM5-KO (n = 5 mice per group). Area under curve (AUC) calculated between 0 to 10 min. ANOVA Fisher’s LSD post hoc.

The cation channel TRPM5 is indispensable for taste receptor signaling in the tongue [12,25], but is also involved in GSIS [26] and STR-mediated insulin secretion [9]. Therefore, we hypothesized that TRPM5 activation is the likely downstream effector between saccharin and GSIS in beta-cells. To address the direct role of TRPM5, we monitored [Ca^2+^]_i_ in dispersed beta-cells isolated from TRPM5-KO. Beta-cells deficient in TRPM5 had an ablated rise in [Ca^2+^]_i_ (Figure 5A) and insulin release (Figure 5B) in response to saccharin. Finally, WT mice transiently increased plasma insulin in response to an IV bolus of saccharin, but this response was partially abolished in TRPM5-KO mice (Figure 5C). Our findings suggest that TRPM5 partly mediates saccharin-induced insulin secretion in beta-cells.

## 4. Discussion

In pancreatic beta-cells, GSIS requires the uptake and metabolism of glucose. Increasing evidence suggests that additional amplifying pathways exist, and that some of them potentiate GSIS by sensing non-glucose nutrients [27]. Some of these nutrients, instead of being metabolized by beta-cells, bind to STR to modulate insulin release [9]. Considering that STR are targets of all classes of AS, it conceivable that these dietary compounds could potentiate insulin secretion. Indeed, it has been known for decades that non-metabolizable AS, such as saccharin, stevioside, and acesulfame K, could stimulate insulin secretion in vitro and in vivo [6,28,29,30,31]. Due to the fact that these studies were performed before the gustatory sensory machinery was characterized [11,32], STR were not implicated. Saccharin is a potent STR ligand, and its consumption has been recently associated with the development of glucose intolerance mediated by changes in gut microbiota [2]. However, our group was not able to replicate these findings in mice or humans [3]. Interestingly, about 90% of ingested saccharin is absorbed in the small intestine and eliminated intact primarily in the urine [33]. Thus, the portion of ingested saccharin that can reach and potentially interact with microbes at the large intestine is very small. Instead, a significant portion of saccharin may reach the blood, targeting various organs.

We hypothesized that, since ingested saccharin can be rapidly absorbed, it may target and activate STR signaling in beta-cells. Consistent with this reasoning, the addition of saccharin during an IGGTT reduced glucose excursions in WT mice and rapidly increased plasma insulin, but it had no effects in T1R2-KO mice. These findings cannot be attributed to sweet taste sensing on the tongue because the IG gavage bypasses the oral cavity. This excludes contributions from the cephalic phase of insulin secretion [34,35]. Nevertheless, STR are expressed in enteroendocrine cells to regulate incretin secretion and glucose absorption [13,36], but the addition of saccharin to an oral glucose challenge did not alter glucagon-like peptide 1 (GLP-1) responses in humans [37]. Here, the direct IP injection of saccharin, to bypass the intestine, increased insulin secretion within minutes in WT mice, but not in T1R2-KO. Notably, in a pioneering study conducted 60 years ago, it was noted that the IP injection of saccharin in overnight-fasted mice caused an unexpected hypoglycemic response 30 min post-injection [38]. These observations are consistent with a rapid increase in plasma insulin during fasting glycaemia. Taken together, these findings are consistent with a direct effect of saccharin on STR expressed on beta-cells, although the use of mice with a whole-body deletion of T1R2 may limit data interpretation. Thus, it is plausible that saccharin stimulates insulin secretion by targeting STR expressed elsewhere. Glucagon can also regulate insulin secretion [39], but we showed, for the first time, that STR are not expressed in alpha-cells. Alternatively, insulin secretion can be regulated by neuronal mechanisms [40]. However, the saccharin effect on insulin secretion persisted in isolated islets, where the neuronal input is absent. The timing of insulin responses to the IP bolus of saccharin does not favor neuronal involvement either. Such a scenario would require saccharin to cross the blood–brain barrier and then effectively initiate such mechanisms. In contrast to our findings in mice here, the addition of saccharin to an OGTT did not alter glucose or insulin responses in humans [37]. However, the saccharin concentration in the human study was 0.3 mg/mL in order to approximate one packet of Sweet n’ Low in a cup. Here, we used 1.0 mg/mL, which is higher, but clearly a physiologically relevant concentration. Thus, the absence of an effect in humans could have been due to the use of a sub-stimulatory dose.

Sweet taste sensing and signaling requires the formation of a T1R2/T1R3 heterodimer [14,41]. Although the mRNA expression of T1r2 and T1r3 has been previously reported in whole mouse islets [8,9], commercially available antibodies targeting STR are not specific. This has prevented the identification of cell types expressing T1R2 and T1R3 in islets. The T1R3 receptor is also required for umami taste (T1R1/T1R3) [11,42], so the presence of the T1R2 receptor is more specific to STR signaling. Therefore, we developed a T1R2 reporter mouse and showed, for the first time, that T1R2 is specifically expressed in the beta-cells, but not in the alpha-cells, of mouse islets. This also confirms mRNA expression data in the MIN6 beta-cell line [8,9] and further validates MIN6 as a reliable tool to explore STR signaling. In the tongue, ligand binding to STR activates PLCβ2, causing the hydrolysis of phosphatidylinositol 4,5-bisphosphate (PIP_2_) to IP_3_ and diacylglycerol (DAG). IP_3_ binding to IP_3_-receptors (IP_3_-Rs) in the ER stimulates calcium release. Calcium then activates TRPM5 channels, allowing sodium influx and cell membrane depolarization. The resulting VDCCs activation leads to calcium influx, and elevated [Ca^2+^]_i_ triggers the exocytosis of ATP [12]. Consequently, loss-of-function of TRPM5 or PLCβ2 eliminates all sweet and umami taste responses in mice [12,43]. This PLC-dependent signaling mechanism was recapitulated in mouse islets in response to the natural sugar, fructose [9]. However, ligand bias is a known feature of GPCRs [44], so we investigated whether saccharin mediated its effects on insulin secretion through a similar mechanism. The knockdown of T1r3 with siRNA caused a reduction in [Ca^2+^]_i_ in response to saccharin compared to scrambled siRNA, further confirming the direct involvement of STR. As with fructose [9], the effects of saccharin on mobilizing intracellular calcium were dependent of VDCCs activation and extracellular calcium influx. Notably, the saccharin-induced rise in [Ca^2+^]i was eliminated at sub-physiological glucose levels, suggesting that adequate glucose concentrations are vital to maintain the necessary membrane depolarization threshold that can be amplified by saccharin to potentiate GSIS.

Using calcium imaging and TIRF microscopy, we also demonstrated that saccharin rapidly activates PLC, preceding the rise in [Ca^2+^]_i_. The activation of PLC persisted independently of glucose availability or VDCCs activation, suggesting that saccharin-induced PLC signaling is direct and upstream of calcium mobilization. Thus, the delayed rise in cAMP shown before in response to saccharin [8] could have been a secondary response. Nevertheless, the heterogeneity of signaling pathways associated with STR stimulation has been demonstrated with other AS, such as sucralose [45]. Munc13-1 is necessary for insulin granule fusion and thus regulates insulin secretion [17,18,22]. Due to the fact that PLC signaling potentiated Munc13-1-mediated exocytotic amplification in neurons [46,47], we hypothesized that Munc13-1 may be a downstream component of saccharin signaling. In MIN6 beta-cells, the activation of STR by the saccharin-induced rapid translocation of Munc13-1 to the plasma membrane is dependent on PLC activity. Indeed, in isolated islets, the inhibition of PLC eliminated the potentiating effects of saccharin on insulin secretion. These data strongly suggest that saccharin-induced insulin responses in beta-cells require the activation of a PLC signaling cascade, leading to insulin exocytosis.

In addition, we also showed that TRPM5 is required for saccharin-induced insulin secretion. These findings agree with previous reports showing that TRPM5 loss-of-function compromised both GSIS and the fructose-induced potentiation of GSIS [9,26]. Notably, the genetic ablation of TRPM5 did not eliminate calcium or insulin responses, suggesting that other channels may account for the residual effects. For instance, TRPM4 is a cation channel functionally related to TRPM5 [48], which also controls insulin secretion [49,50]. Thus, TRPM4 may partially compensate for the absence of TRPM5 activity in our findings. Consequently, TRPM channel activation is likely required for membrane depolarization and calcium influx amplification.

In summary, using genetic and pharmacological approaches, we show that the ingestion of saccharin can stimulate insulin secretion in the presence of glucose. These effects are dependent on STR that are specifically expressed in beta-cells. Like other sweeteners, saccharin rapidly induces the PLC-dependent activation of the cation TRPM channels, leading to calcium influx, membrane depolarization, and the vesicular exocytosis of insulin. These data shed light on the acute physiological effects of saccharin ingestion, providing a foundation for understanding potential effects of chronic saccharin use. In addition, our studies are proof-of-concept that targeting STR on beta-cells may be a feasible therapeutic strategy to enhance insulin secretion.
